# Risk-Taking Behaviors Among Adolescents in Single-Mother Versus Two-Parent Households in India: A Cross-Sectional Comparative Study

**DOI:** 10.7759/cureus.101884

**Published:** 2026-01-20

**Authors:** Siddharth Dutt, Bangalore Roopesh, Navaneetham Janardhana

**Affiliations:** 1 Psychology, National Institute of Mental Health and Neurosciences, Bangalore, IND; 2 Clinical Psychology, National Institute of Mental Health and Neurosciences, Bengaluru, IND; 3 Psychiatric Social Work, National Institute of Mental Health and Neurosciences, Bangalore, IND

**Keywords:** adolescents, children of single mothers, children of single parent, children with both parents, risk-taking

## Abstract

Background

Adolescence is a developmental period marked by heightened vulnerability to risk-taking behaviors due to ongoing neurobiological maturation and increasing exposure to social and environmental influences. Family context represents a key ecological determinant of adolescent risk trajectories. Although children of single mothers constitute a growing demographic in India, comparative evidence on their risk-taking behaviors relative to adolescents living with both parents remains limited. This study examined differences in risk-taking behaviors between these two family contexts in an urban Indian setting.

Methods

A cross-sectional comparative design was employed with a convenience sample of 119 adolescents aged 12-18 years, which included children of single mothers (CSM; n=59) and children living with both parents (CLBP; n=60). Risk-taking behaviors were assessed using a semi-structured interview schedule covering mobility-related risks, substance use-related behaviors, school engagement, and conduct-related behaviors. Group differences were examined using chi-square or Fisher's exact tests. Adjusted logistic regression analyses, including penalized models where appropriate, were conducted for selected outcomes while controlling for age, sex, and educational attainment.

Results

Adolescents from single-mother households demonstrated significantly lower school attendance and higher prevalence of running away from home and gang involvement compared to adolescents living with both parents. Adjusted analyses indicated that CSM status was independently associated with lower odds of school attendance and higher odds of gang involvement. In contrast, CLBP adolescents showed significantly higher engagement in opportunity-driven mobility risks, particularly underage two-wheeler riding without a license. Substance use behaviors were infrequent in both groups and did not differ significantly.

Conclusion

Adolescent risk-taking is not uniformly elevated among children of single mothers but is concentrated in domains reflecting educational disengagement and psychosocial vulnerability. Findings underscore the need for context-sensitive interventions that strengthen educational continuity and social support for adolescents in single-mother households.

## Introduction

Risk-taking behavior has been defined as behaviors that increase the likelihood of adverse psychological, social, or health consequences [1]. Such behaviors include delinquency, antisocial behavior, violence, substance use, early and unprotected sexual activity, and other health-compromising actions [2]. Engagement in these behaviors during adolescence has been linked to poorer mental health, academic failure, social exclusion, and increased morbidity and mortality in adulthood.

Multiple contextual and individual risk factors contribute to the emergence of risk-taking behaviors, including low socioeconomic status, ethnicity and race, limited parental education and involvement, weak family support systems, adverse peer influences, intellectual or learning disabilities, and temperament-related vulnerabilities.

Neurodevelopmental research provides important insights into why adolescence represents a particularly sensitive period for risk-taking behavior. Studies of adolescent brain development indicate that the prefrontal cortex, particularly regions involved in decision-making and impulse control, such as the ventromedial prefrontal cortex, is still maturing during adolescence [3]. Experimental studies have shown that during risk-taking tasks, adolescents under-recruit neural systems responsible for cognitive control, including the prefrontal cortex and anterior cingulate cortex, relative to adults [4]. This neurodevelopmental imbalance contributes to heightened sensitivity to rewards and reduced capacity for self-regulation.

Social and relational factors play a critical protective role in shaping adolescent risk trajectories. Research has consistently shown that parent-adolescent relationships characterized by warmth, trust, positive communication, and nurturance are associated with lower rates of substance use, including tobacco use [5-7]. Similarly, higher-quality school environments have been linked to reduced engagement in risk-taking behaviors [8]. However, marginalized children often attend poorly resourced schools and experience inappropriate caregiving relationships, limiting exposure to these protective influences.

Peer relationships also exert a significant influence on adolescent risk-taking. Studies indicate that peer group composition matters, with religiously oriented peer groups associated with lower risk-taking behaviors, while certain youth-based peer groups may increase engagement in risky activities [9]. Decisions made in peer contexts are more likely to favor immediate rewards over long-term consequences, particularly during adolescence when sensation-seeking tendencies are heightened.

Family structure and parental monitoring further shape risk trajectories. Adolescents living with a single parent or experiencing family disruption have shown variability in risk outcomes depending on the quality of parent-child relationships. High levels of trust, open communication, and effective parental monitoring have been associated with delayed initiation of sexual activity and greater use of protective sexual behaviors [10-12]. Mentorship, whether from adults or peers, has also been shown to reduce risky sexual behaviors [13,14]. Marginalized families, however, often face economic and social constraints that limit consistent supervision and monitoring.

Family context constitutes a core ecological system shaping adolescent development. Single-mother households are frequently exposed to cumulative and intersecting adversities that heighten social, economic, and psychological vulnerability for both caregivers and children. Extensive evidence indicates that single mothers are disproportionately affected by poverty, employment instability, and housing insecurity, largely due to gendered wage gaps, limited access to stable employment, and the absence of a co-resident income contributor [15]. These structural disadvantages often co-occur with elevated caregiving burdens, reduced social support, and chronic stress, creating conditions of cumulative risk rather than isolated hardship [16].

From a psychosocial perspective, prolonged exposure to economic strain and role overload has been associated with higher rates of maternal depression, anxiety, and parenting stress in single-mother households, which in turn can affect parenting practices and parent-child relationships [17]. Children raised in such contexts may face increased risks of emotional and behavioral difficulties, not as a consequence of single parenthood per se, but due to the clustering of adversities such as financial deprivation, neighborhood disadvantage, and reduced access to supportive services [18].

Children in single-mother families are not "destined" for risk-taking, but the context increases the chances. With one person managing both work and caregiving, which is often associated with financial strain and time constraints, this can reduce supervision and predictable routines, increasing exposure to delinquent peers and opportunities to try substances, early sex, or other hazards [19,20]. Financial hardship can also heighten caregiver stress and depressive symptoms, which may weaken warmth and consistency in parenting, a pathway linked to externalizing behavior and later substance use [21]. Family transitions that often precede single motherhood (separation, conflict, residential moves) can disrupt attachment security and school engagement, nudging adolescents toward sensation-seeking and peer approval as coping [22]. However, the above does not apply to all single-parent households. Many single mothers do have stable incomes, provide protective environments, and supportive kin networks. Along with this, good schools can reduce risk-taking behaviors by increasing monitoring, belonging, and access to prosocial activities [23].

India-specific research is needed to test whether, how, and when single-mother contexts amplify or buffer these risks, and to inform targeted school, community, and social-protection interventions. Hence, this study compares adolescent risk-taking behaviors across single-mother and two-parent households.

## Materials and methods

Study design and participants

The study employed a cross-sectional design to compare risk-taking behavior across two groups: children of single mothers (CSM) and children living with both parents (CLBP). A convenience sampling strategy was used. Ethical approval was obtained from the Institutional Ethics Committee prior to data collection. Inclusion criteria for both children of single mothers and children living with both parents comprised boys and girls aged 12-18 years, with a minimum educational attainment of third standard, and the ability to understand and communicate in English, Kannada, or Hindi. Exclusion criteria included adolescents with intellectual disability, as determined through clinical evaluation, which could interfere with comprehension of the interview schedule or provision of informed assent.

The sample comprised adolescents of both genders, aged 12-18 years, including 59 CSM and 60 CLBP. The sample size was estimated using a two-proportion comparison framework. Assuming a medium effect size (w=0.30), α=0.05, and power (1-β)=0.80, the minimum required sample size was 88 participants. Allowing for non-response and subgroup comparisons, a final sample of 119 adolescents was recruited.

Tool description

A semi-structured interview guide for risk-taking behavior was developed by the researchers for the present study (see Appendix 1). The guide assessed engagement in behaviorally specific risk-taking activities across four domains: mobility-related risks, substance use-related risks, school engagement, and conduct-related behaviors. Items were coded dichotomously (0=behavior absent, 1=behavior present), consistent with prior community-based adolescent risk research focusing on low-base-rate behaviors. The schedule was administered by the first author (Siddharth Dutt), which minimized inter-interviewer variability. Content validity was established through expert review by 15 professionals in child and adolescent mental health, and pilot testing was conducted on eight adolescents to check clarity, cultural appropriateness, and sensitivity of items. Adolescents found the content of the schedule clear and appropriate for their age group. This can be attributed to the large number of experts who had gone through the interview schedule thoroughly. Given the exploratory and context-specific nature of the study, the tool was intended to capture prevalence and patterns of risk behaviors rather than generate psychometric scale scores.

Procedure

The semi-structured interview schedule was developed through a systematic and iterative process. An initial pool of items was generated based on an extensive review of empirical literature on adolescent risk-taking behavior and theoretical models of developmental risk. These items were organized into thematic domains covering substance use-related risks, mobility-related risks, school engagement, and conduct-related behavior. The draft schedule was reviewed by experts in child and adolescent mental health to establish content validity and cultural relevance. Subsequently, pilot interviews were conducted to assess clarity, sensitivity, and comprehensibility, leading to refinement of wording, sequencing, and probes.

For the CSM group, NGO-run shelter homes were contacted, and following institutional permission, eligible adolescents were approached for participation. For the CLBP group, three schools and two colleges were contacted; one semi-urban school and one urban college provided permission. Written informed consent was obtained from the respective institutions and parents or guardians, along with written informed assent from all participating adolescents. Similarly, adolescents who could understand and respond in English, Kannada, or Hindi were included in the study. The socio-demographic data sheet prepared for the study and the semi- structured interview schedule for risk-taking behavior were administered individually to each adolescent.

Data collection with adolescents from stigmatized and structurally vulnerable backgrounds required careful attention to power dynamics, confidentiality, and non-judgmental engagement. Interviews were conducted in a supportive manner, emphasizing voluntariness and psychological safety, particularly for participants residing in institutional care settings.

Data analysis

Data were analyzed using a combination of descriptive and inferential statistical techniques to examine patterns of risk-taking behavior across the two groups. Data were analyzed using IBM SPSS Statistics for Windows, Version 26.0 (IBM Corp., Armonk, USA). Frequencies and percentages were computed for categorical variables across domains of substance use-related risks, mobility-related risks, school engagement, and conduct-related behavior, while means and standard deviations were calculated for continuous variables such as frequency of behaviors. Group differences for categorical variables were examined using chi-square tests, and a t-test was employed for continuous variables to assess between-group differences. Statistical significance was set at p<0.05. All analyses were performed to identify both overall trends and domain-specific variations in risk-taking behavior among the groups. Additionally, adjusted logistic regression analyses, including penalized models where appropriate, were conducted for selected outcomes to examine group differences while controlling for age, sex, and educational attainment.

## Results

The comparison of sociodemographic profiles between CSM (n=59) and CLBP (n=60) was in terms of risk-taking behaviors across mobility-related risks, substance use-related behaviors, school engagement, and conduct-related behaviors is presented in Table 1. There was a statistically significant difference in age between the CSM and CLBP groups, with adolescents in the CSM group being older on average. However, no significant difference was observed in years of education, indicating that both groups were comparable in educational attainment. Gender distribution did not differ significantly between groups. Place of stay differed by design, with most CSM participants residing in NGO settings, whereas all CLBP participants resided at home.

**Table 1:** Comparison of sociodemographic profiles between CSM and CLBP CSM - children of single mothers; CLBP - children living with both parents

Variable	Group	n	Mean (SD) or n	Statistic	p-value
Age (years)	CSM	59	15.4 (2.1)	t=4.24	<0.001
	CLBP	60	13.9 (1.7)		
Education attainment	CSM	59	8.5 (2.1)	t=-0.6	0.51
	CLBP	60	8.8 (1.7)		
Gender	CSM	59	Male: 29; female: 30	χ²=2.01	0.16
	CLBP	60	Male: 20; female: 40		
Place of stay	CSM	59	Home: 13; NGO: 46	n/a	n/a
	CLBP	60	Home: 60; NGO: 0		

Riding a two-wheeler was more frequently reported among CLBP (n=33, 55%) compared with CSM (n=23, 39%), and this difference was statistically significant (Table 2). Other mobility-related behaviors were uncommon and did not differ significantly between groups, including riding triple, performing stunts, and riding while intoxicated.

**Table 2:** Comparison of risk-related behaviors in CSM and CLBP groups CSM - children of single mothers; CLBP - children living with both parents

Risk-taking behavior	CSM (n=59)	CLBP (n=60)	Test statistic	p-value
Riding a two-wheeler	23 (39%)	33 (55%)	χ²=2.45	0.117
Riding triple	9 (15%)	8 (13%)	χ²=0.00	0.97
Riding intoxicated	1 (2%)	0 (0%)	n/a	n/a
Performing stunts	7 (12%)	4 (7%)	χ²=0.44	0.508
Smoking cigarettes	6 (10%)	1 (2%)	χ²=0.04	0.061
Chewing tobacco	4 (7%)	0 (0%)	n/a	n/a
Consuming alcohol	2 (3%)	0 (0%)	n/a	n/a
Played cards	2 (3%)	0 (0%)	n/a	n/a
Attending school	47 (80%)	60 (100%)	χ²=11.42	<0.001
Run away from home	19 (32%)	1 (2%)	χ²=17.72	<0.001
Staying outside late	14 (24%)	15 (25%)	χ²=0.00	1
Street fights	4 (7%)	1 (2%)	χ²=2.23	0.207
Fights in school	4 (7%)	4 (7%)	χ²=0.00	0.9
Gang activity	5 (8%)	0 (0%)	χ²=7.1	0.027

Substance use-related behaviors were reported at relatively low frequencies in both groups. Cigarette smoking was descriptively higher in CSM (n=6, 10%) than in CLBP (n=1, 2%), but the group difference did not reach statistical significance. Chewing tobacco, alcohol consumption, and playing cards were infrequently endorsed (Table 2).

Statistically significant group differences emerged for school engagement indicators. School attendance was lower among CSM (n=47, 80%), compared to universal attendance in the CLBP group (n=60, 100%). Running away from home was substantially more common among CSM (n=19, 32%) than among CLBP (n=1, 2%), reaching a statistically significant difference between groups (Table 2).

Most conduct-related behaviors did not differ significantly between groups, including staying outside late, street fights, and fights in school (see Table 2). However, gang activity was reported only in the CSM group (n=5, 8%) and not in CLBP (n=0), representing a statistically significant difference.

Adjusted logistic regression analysis was conducted for theoretically salient outcomes with sufficient variability: riding a two-wheeler, running away from home, school attendance, and gang activity (see Table 3). Family context (CSM vs CLBP) was the primary independent variable. Age, sex, and educational level were included as covariates. Penalized logistic regression with bootstrap confidence intervals was used for school attendance and gang activity due to outcome separation or low event frequency.

**Table 3:** Adjusted logistic regression analysis of adolescent risk-taking behaviors (CSM vs CLBP) † Adjusted odds ratios (AOR) were estimated using logistic regression. Penalized logistic regression with bootstrap confidence intervals was used for school attendance and gang activity due to outcome separation or low event frequency. Models are adjusted for age, sex, and educational attainment; outcomes coded as binary (1=yes, 0=no). *** p<0.001; ** p<0.01, * p<0.05 CSM - children of single mothers; CLBP - children living with both parents

Predictor	Riding two-wheeler, AOR (95% CI)	Running away, AOR (95% CI)	School attendance†, AOR (95% CI)	Gang activity†, AOR (95% CI)
Family context: CSM vs CLBP	0.23 (0.09-0.59)**	8.38 (0.95-73.91)	0.38 (0.22-0.64)**	3.18 (1.50-5.53)*
Age (years)	1.28 (0.97-1.70)	1.83 (1.26-2.65)**	0.66 (0.57-0.79)***	0.79 (0.69-0.91)**
Education level	0.91 (0.70-1.18)	0.53 (0.36-0.78)**	2.34 (1.81-3.11)***	0.97 (0.73-1.16)
Sex: male vs female	5.69 (2.60-12.45)***	1.76 (0.58-5.35)	0.54 (0.27-1.14)	2.32 (0.91-4.53)

After adjustment, CSM (vs CLBP) showed lower odds of riding a two-wheeler and lower odds of school attendance, alongside higher odds of gang activity; the adjusted association with running away from home was large in magnitude but not statistically significant. Older age was associated with higher odds of running away and lower odds of school attendance. Male sex strongly predicted riding a two-wheeler.

## Discussion

In the present study, risk-taking behavior among adolescents was examined using a semi-structured interview schedule covering mobility-related risks, substance use-related risks, school engagement, and conduct-related behaviors. The combined use of descriptive statistics, bivariate analyses, and adjusted logistic regression enabled a nuanced assessment of both unadjusted group differences and associations that persisted after controlling for key demographic confounders. By focusing specifically on children of single mothers and children living with both parents, the findings offer insight into how family structure and associated caregiving contexts shape adolescent risk trajectories in the Indian setting.

Mobility-related risk behaviors emerged as a prominent area of differentiation between the two groups. Despite Indian legal restrictions prohibiting riding a two-wheeler below 18 years of age, more than half of adolescents in the CLBP group reported riding a two-wheeler, compared to just over one-third of those in the CSM group. Adjusted regression analyses confirmed that adolescents from single-mother households had significantly lower odds of riding a two-wheeler even after accounting for age, sex, and educational attainment. This pattern suggests that higher engagement in underage riding among CLBP adolescents is likely driven by differential access and opportunity rather than a greater inclination toward risk [24,14]. Adolescents living with both parents may be more likely to reside in households where two-wheelers are owned and where parental norms tacitly permit early riding, either for convenience or skill acquisition. Indian road-safety literature similarly emphasizes that adolescent exposure to traffic risk often follows vehicle availability and permissive family norms rather than deliberate deviance [24].

Other mobility-related behaviors, including triple riding, performing stunts, and riding while intoxicated, did not differ significantly between groups. These behaviors were infrequently reported and appear to reflect normative adolescent experimentation and peer-influenced thrill-seeking rather than structural or family-specific vulnerability [23,24]. The absence of group differences suggests that certain risky riding practices may be embedded within broader youth culture, shaped by peer norms and media exposure, rather than by household structure alone [25].

Substance use-related behaviors were generally uncommon in both groups, with no statistically significant differences observed for cigarette smoking, tobacco chewing, or alcohol consumption. Low event frequencies precluded inclusion of these variables in regression analyses. Notably, the prevalence of substance use in this sample was lower than national estimates reported in surveys such as the Global Youth Tobacco Survey and other national adolescent health reports [26,27]. Although descriptively higher rates of smoking and tobacco use were observed among CSM adolescents, these differences did not reach statistical significance. This pattern aligns with evidence suggesting that risk in single-mother households is shaped less by family structure itself and more by contextual stressors such as economic strain, caregiving burden, and reduced supervision, which may or may not translate into substance use depending on opportunity and monitoring [15].

School engagement emerged as one of the most salient domains distinguishing the two groups. While school attendance was universal among adolescents living with both parents, attendance was significantly lower among those from single-mother households. School disengagement during adolescence has been consistently associated with increased vulnerability to behavioral and psychosocial risks, including delinquency, substance use, and poor mental health outcomes, particularly in contexts marked by cumulative socioeconomic adversity [6,14,20]. Importantly, adjusted and penalized regression analyses indicated that CSM adolescents had substantially lower odds of attending school even after controlling for age, sex, and educational attainment. This finding highlights a specific educational vulnerability among children of single mothers that cannot be explained solely by demographic differences. In the Indian context, single mothers often face compounded economic pressures, time constraints, and limited social support, which may reduce their capacity to monitor schooling, manage absenteeism, or buffer adolescents from academic disengagement. Disruptions related to parental separation, bereavement, or migration may further undermine educational continuity.

Running away from home was also markedly more prevalent among CSM adolescents. Although adjusted odds remained large but statistically imprecise due to low event counts, older age and lower educational attainment independently increased the likelihood of runaway behavior. Contemporary literature increasingly conceptualizes running away as a coping response to cumulative stressors rather than as impulsive deviance [22-24]. The convergence of school disengagement, caregiving strain, and limited emotional or instrumental support may place adolescents in single-mother households at particular risk of seeking escape from stressful home environments.

Conduct-related behaviors showed mixed patterns. Staying outside late did not differ significantly between groups, reflecting a normative developmental shift toward peer-oriented socialization during adolescence [24]. Street fights and school-based fights were relatively uncommon and did not show significant group differences. In contrast, gang activity, although infrequent overall, was reported only among CSM adolescents and remained significantly associated with single-mother household status in penalized regression analyses. Gang involvement during adolescence has been linked to unmet needs for supervision, belonging, and social support, particularly in contexts characterized by weakened family monitoring and cumulative environmental risk [25,14,17]. While these findings should be interpreted cautiously due to small numbers, they are consistent with the literature linking gang involvement to unmet needs for belonging, protection, and identity in contexts characterized by reduced supervision and social support [24,25].

Overall, the findings underscore that adolescent risk-taking in the Indian context is not uniformly elevated among children of single mothers across all domains. Rather, specific vulnerabilities emerge in areas related to educational engagement, psychosocial distress, and antisocial affiliation, while other risks appear shaped primarily by opportunity structures and normative developmental processes. These results highlight the importance of moving beyond simplistic assumptions about family structure and instead focusing on modifiable contextual factors [14,15,20] such as educational support, caregiver burden, and community-based scaffolding to mitigate risk among adolescents from single-mother households.

Strengths of the study

A major strength of this study lies in its comparative design, which enabled direct examination of differences between children of single mothers and those living with both parents, rather than treating family structure as a homogeneous variable. This approach allowed for clearer identification of specific vulnerabilities and protective patterns associated with each family context.

The use of a semi-structured interview schedule facilitated the collection of behaviorally specific and contextually grounded data across multiple domains of adolescent risk. The incorporation of adjusted and penalized logistic regression analyses enhanced analytic rigor by accounting for key confounders such as age, sex, and educational attainment, thereby strengthening the validity of observed associations.

Additionally, recruitment from real-world settings such as schools, colleges, and NGO-linked residential contexts enhanced ecological validity and relevance for applied and policy-oriented interpretation. The study was further strengthened by its grounding in an integrative framework drawing on developmental, social, and ecological perspectives, allowing for theoretically informed interpretation of both normative and maladaptive risk-taking behaviors.

Limitations

Several limitations warrant consideration. First, the cross-sectional design limits causal inference, and the temporal ordering between family context and risk-taking behaviors cannot be established. Longitudinal studies are required to examine developmental trajectories and causal pathways.

Second, the use of convenience sampling may restrict generalizability, particularly to adolescents not engaged with educational or institutional settings. Third, reliance on self-reported risk behaviors introduces the possibility of recall and social desirability bias, especially for illegal or socially sanctioned behaviors.

Fourth, certain outcomes were characterized by low event frequencies, necessitating the use of penalized regression models and cautious interpretation of effect estimates. Finally, the study did not include direct measures of parenting practices, peer influences, neighborhood characteristics, or caregiver mental health, all of which may mediate or moderate the relationship between family structure and adolescent risk-taking. Future research incorporating multi-informant and multi-level designs would strengthen explanatory depth and intervention relevance.

Finally, a key limitation is potential selection bias arising from the CSM sample being largely recruited from NGO-run shelter homes, which may over-represent families experiencing significant psychosocial adversity and may not reflect community-dwelling single-mother households. This service-linked, partially institutionalized context could influence both exposure and reporting of risk behaviors, thereby limiting the generalizability of CSM-CLBP comparisons.

Future research

Future research should employ longitudinal designs to examine developmental trajectories of risk-taking among adolescents from diverse family structures. Incorporating multi-informant assessments, including caregiver and teacher reports, and measuring parenting practices, peer networks, and neighborhood characteristics would enhance explanatory depth. Intervention-focused studies evaluating school-based retention programs and community support models for single-mother households are particularly warranted in the Indian context.

## Conclusions

The present study offers a focused comparative examination of risk-taking behaviors among adolescents from two family contexts: children of single mothers and children living with both parents. By integrating descriptive analyses with adjusted logistic regression models, the study demonstrates that observed group differences cannot be explained solely by age, sex, or educational attainment, underscoring the independent influence of family context on adolescent risk trajectories. Adolescents from single-mother households emerged as a particularly vulnerable group across select domains of risk. Lower school attendance, higher prevalence of running away from home, and greater likelihood of gang involvement characterized this group, even after adjustment for key demographic factors. These findings suggest that caregiving strain, economic pressures, and reduced supervisory capacity commonly associated with single-mother households may heighten vulnerability to psychosocial and behavioral risks during adolescence. In contrast, adolescents living with both parents demonstrated higher engagement in opportunity-driven mobility risks, particularly underage two-wheeler riding, highlighting the role of access, availability of vehicles, and permissive household norms rather than psychosocial adversity per se. Taken together, the findings emphasize that adolescent risk-taking is shaped by the interaction of caregiving capacity, educational engagement, and opportunity structures within the family environment. Prevention and intervention efforts should therefore move beyond uniform, deficit-based approaches and prioritize context-sensitive strategies, with particular attention to adolescents in single-mother households who may remain underserved by existing school- or community-based support systems.

## Appendices

Appendix 1: Interview guide for risk-taking behaviour

Administered for adolescents between 12-18 years.

This interview will focus on the topic of risk-taking behaviors. During this interview, I will be asking you a few questions related to this subject. Risk-taking behaviors refer to actions or activities that involve a certain level of risk or uncertainty, where engaging in such behaviors may lead to serious adverse or negative consequences. These consequences can affect an individual's physical health, social, and psychological, or emotional aspects and well-being.

Mobility-related risk behaviour. (Based on the response, further clarification can be done).

1. Have you ever ridden a motorbike/scooter/moped? Yes/No

2. Have you ever ridden triple (three people riding on a motorbike/scooter/moped)? Yes/No

3. Have you ever ridden (motorbike/scooter/moped) intoxicated? Yes/No

4. Have you ever done stunts on the motorbike/scooter/moped (e.g., Wheelie, Stoppie)? Yes/No

Substance use-related behaviors. (Based on the response, further clarification can be done). 

5. Have you ever smoked cigarettes or beedi or other substances? Yes/No

6. Have you ever chewed tobacco leaves? Yes/No

7. Have you ever consumed alcohol? Yes/No

Gambling. (Based on the response, further clarification can be done).

8. Have you ever played cards (or other related activities) for money? Yes/No

School engagement. (Based on the response, further clarification can be done).

9. Do you regularly attend school? Yes/No

10. Have you ever stayed late outside home (e.g., after 9 pm)? Yes/No

Conduct-related behaviors. (Based on the response, further clarification can be done).

11. Have you ever run away from home? Yes/No

12. Have you ever got into street fights? Yes/No

13. Have you ever got into any gang activity? Yes/No
